# Clinical and genomic characterization of Influenza A co-infection with SARS-CoV-2 and Influenza B: a respiratory surveillance study in Assam, India

**DOI:** 10.1038/s41598-026-55080-0

**Published:** 2026-06-12

**Authors:** Neelanjana Sarmah, Aktarul Islam Siddique, Mousumi Dutta, Aniruddha Jakharia, Nargis Bali, Neetu Vijay, Nivedita Gupta, Suman Kanungo, Biswajyoti Borkakoty

**Affiliations:** 1 Regional Viral Research and Diagnostic Laboratory (Regional VRDL), ICMR–Regional Medical Research Centre (RMRC), NE Region, Bokel, Dibrugarh, Assam 786010 India; 2 Department of Clinical Microbiology, Sher-i-Kashmir Institute of Medical Sciences, Soura, Srinagar, 190011 India; 3 Indian Council of Medical Research, New Delhi, India; 4 Communicable Diseases Division, Indian Council of Medical Research, New Delhi, India

**Keywords:** Co-infection, Influenza A (H3N2), Influenza B (Victoria), SARS-CoV-2, ARI, SARI, Computational biology and bioinformatics, Diseases, Genetics, Microbiology

## Abstract

Influenza and SARS-CoV-2 are the primary contributors to seasonal respiratory infections and frequently co-circulate, creating significant health challenges. The present respiratory surveillance study was conducted in Dibrugarh, Assam, India from January 2025 to August 2025 to investigate the genomic characteristics of circulating viruses and identify potential co-infections. Overall, 4,948 respiratory samples were screened using multiplex real-time PCR, followed by subtyping of Influenza A and Influenza B. Next-generation sequencing (NGS) was performed in selected positives of SARS-CoV-2 and Influenza A. Genomic analysis included mutational profiling, phylogenetic analysis and N-glycosylation site prediction using bioinformatics tools. Two co-infection cases were detected: one involving Influenza A (H3N2) with SARS-CoV-2 (Omicron XFG lineage) and another involving Influenza A (H3N2) with Influenza B (Victoria lineage). Both patients experienced mild illness without hospitalisation. NGS revealed that the Influenza A (H3N2) viruses belonged to clade 3C.2a1b.2a.2a.3a.1 while SARS-CoV-2 sequence was classified under the Omicron XFG lineage. Mutational analysis of the HA gene showed several amino acid differences compared to the reference vaccine strain A/Darwin/6/2021. N-glycosylation analysis predicted conserved sites at positions 79, 181, 262, and 301 in all strains along with an additional predicted site at position 110 in both co-infection cases. Although the co-infection cases presented with mild clinical manifestations, the observed genomic variations indicate a potential role of co-infecting viruses in shaping viral evolution. Given the limited genomic data available from Northeast India, the study underscores the need for sustained large scale follow up and genomic surveillance to monitor emerging mutations and target future vaccine strategies.

**Supplementary Information:**

The online version contains supplementary material available at https://doi.org/10.1038/s41598-026-55080-0.

## Introduction

Influenza viruses and SARS-CoV-2 are the primary contributors to seasonal respiratory illnesses, significantly impacting healthcare management and treatment. According to CDC records, 9.3–41 million flu cases have been reported, leading to 120,000 to 710,000 hospitalisations and 6,300 to 52,000 annual deaths in the USA from 2010 to 2025^1^. Influenza viruses are members of the family Orthomyxoviridae with a genome size of 13.5–14.6 kb^2^. Among the four groups of Influenza viruses (Influenza A-D), Influenza A is primarily responsible for causing pandemics and Influenza B is solely associated with seasonal epidemics across the globe^3,4^. Influenza B viruses are restricted to humans, lack animal reservoirs of comparable diversity, and are associated solely with seasonal epidemics rather than pandemics. The Influenza virus genome comprises 8 segments with hemagglutinin (HA) and neuraminidase (NA) being the major genes^5^. The genome of SARS-CoV-2 possesses a positive-sense ssRNA with a genome size of approximately 30kb^6^. WHO COVID-19 dashboard data showed that as of 16th November, 2025, a total of 778 million SARS-CoV-2-positive cases were detected worldwide, with 7.1 million deaths^7^. Since its emergence in 2019 at Wuhan, China, multiple variants of this virus have been reported globally with significant structural and genomic changes^8^.

Co-infection refers to the concurrent infection of a single host by two or more pathogens due to shared transmission routes^9^. Co-infections with respiratory viruses might alter the disease symptoms with increased complications^10^. Of note, Influenza and SARS-CoV-2 possess a similar trend of droplet transmission for infecting the upper and lower respiratory tract, leading to identical clinical representation of symptoms^11^. Furthermore, the simultaneous seasonal circulation of these viruses increases the likelihood of co-infections^12^. Multiple reports have documented co-infections of Influenza A combined with SARS-CoV-2 and Influenza B^13–16^. Although Influenza A and B co-infections are less common, but may cause complications in immunocompromised individuals^11,14^. Co-infections of SARS-CoV-2 with Influenza A subtypes H3N2 and H1N1 have been reported previously with a spectrum of clinical severity ranging from mild to severe^13,17^. Previously published studies indicated that SARS-CoV-2 and Influenza co-infections may lead to higher severity and mortality compared to other respiratory pathogens^18–21^.

Studies on respiratory viral co-infections, particularly involving Influenza A, Influenza B, and SARS-CoV-2 remain limited in India, with even fewer reports from Northeast India^13,22^. Moreover, molecular and genomic investigations of such co-infections including analysis of lineage patterns, mutation profiles and predicted glycosylation site are largely lacking from this region. To address this gap, the present study investigated the clinical, demographic and genomic profiling of the two co-infection cases: Influenza A (H3N2) with SARS-CoV-2 and Influenza A (H3N2) with Influenza B (Victoria). These cases were identified through routine surveillance of suspected acute respiratory infections (ARI) and Paediatric severe acute respiratory infections (SARI) cases in Dibrugarh, Assam, India. The present study thus represented one of the first detailed genomic reports of such co-infections from Northeast India.

## Results

### Patient demographic and clinical characteristics

During the study period (1st January 2025 to 31st August 2025), overall, 4948 ARI/ Paediatric SARI samples were tested in the laboratory. The mean age of the study group was 13.6 years (SD ± 17.2 years), comprising 86.1% of ARI and 13.8% of Paediatric SARI cases. The age distribution was compared group wise and found to be statistically significant with a p value < 0.0001. The mean duration of illness of the study participants was found to be 3.4 days (SD ± 6.7). Fever was the predominant symptom reported in the majority of cases, followed by cough, headache, and rhinorrhoea. Other symptoms included sore throat, chills, breathlessness, vomiting, and myalgia. Symptoms like nausea, rigor and altered sensorium were less common.

### Detection of Influenza (A and B) and SARS-CoV-2 in ARI/SARI samples

Of the tested samples, 12.3% (611/4948) were positive for Influenza A, 2.6% (128/4948) were positive for Influenza B, and 4.1% (204/4948) were positive for SARS-CoV-2. Since, Influenza A has been detected as the most predominant virus in our study, clinical and demographic parameters were analysed with respect to Influenza A positivity as presented in Table 1. The association between demographic and clinical variables and Influenza A positivity was assessed using odds ratios (OR) along with 95% confidence intervals (CI). Influenza A positivity showed significant association with age (*p<0.001*)  , patient type (*p <0.001*), duration of illness (*p*=*0.008*) and seasonal pattern (*p<0.001*) as shown in Table 1. However, the gender was not significantly associated with Influenza A infection (*p* = *0.065*). Taking 0–5 years as the reference age group, the highest odds were observed among children aged 6–15 years (OR = 3.10; 95% CI: 2.50–3.84), followed by individuals aged 16–45 years and ≥ 46 years (Table 1). Outdoor cases showed significantly higher odds of Influenza A positivity compared to indoor cases (OR = 3.31; 95% CI: 2.29–4.80). A strong seasonal pattern of Influenza A infection was observed with the increased positive cases during the summer months. Compared to winter, the odds of Influenza A positivity were significantly higher in spring (OR = 1.90; 95% CI: 1.20–3.03) and markedly elevated in summer (OR = 12.99; 95% CI: 8.70-19.45) as shown in Table 1. The relationship between major clinical symptoms and Influenza A positivity was also determined and presented in supplementary Table 1. Individuals with fever were significantly more likely tested positive for Influenza A exhibiting nearly a fivefold increase in odds compared with those without fever (OR = 5.03; 95% CI: 3.26–7.76; *p* *< 0.001*) as shown in supplementary Table 1. Further, fever was also found to be significantly associated with Influenza B (*p* *= 0.003*) and SARS-CoV-2 (*p <0.001*) infection.

**Table 1:** Demographic and clinical characteristics of the enrolled subjects for Influenza A PCR.

Variable	Total (*N*)	Influenza A positive *N* (%)	Influenza A negative *N* (%)	Odds ratio	95% CI	*P* value
**Gender**						
Male	2468	323 (13.1)	2145 (86.9)	1.0	Ref	0.065 ^Ψ^
Female	2477	288 (11.6)	2189 (88.4)	0.874	0.73–1.02	
**Age in years**						
0–5 years	2425	199 (8.2)	2226 (91.8)	1.0	Ref	
6–15 years	903	196 (21.7)	707 (78.3)	3.10	2.50–3.84	<0.001*
16–45 years	1256	171 (13.6)	1085 (86.4)	1.76	1.42–2.19	
46 & above	339	43 (12.7)	296 (87.3)	1.62	1.14–2.31	
**IPD/OPD**						
Indoor	678	31 (4.6)	647 (95.4)	1.0	Ref	<0.001 ^Ψ^
Outdoor	4212	577 (13.7)	3635 (86.3)	3.31	2.29–4.80	
**Duration of illness**						
0–7 days	4723	595 (12.6)	4128 (87.4)	1.0	Ref	0.008 ^Ψ^
7 days and above	191	13 (6.8)	178 (93.2)	0.50	0.28–0.89	
**Seasonal pattern**						
Winter	1162	26 (2.2)	1136 (97.8)	1.0	Ref	
Spring	1509	63 (4.2)	1446 (95.8)	1.90	1.20–3.03	<0.001*
Summer	2277	522 (22.9)	1755 (77.1)	12.99	8.70-19.45	

A p-value < 0.05 considered significant, ^Ψ^ Based on Fisher’s Exact Test, *Based on Pearson Chi Square Test. Reference category is indicated as “Ref”. Seasonal classification: Winter (January-February), Spring (March-May), Summer (June-August).

**Fig. 1:**
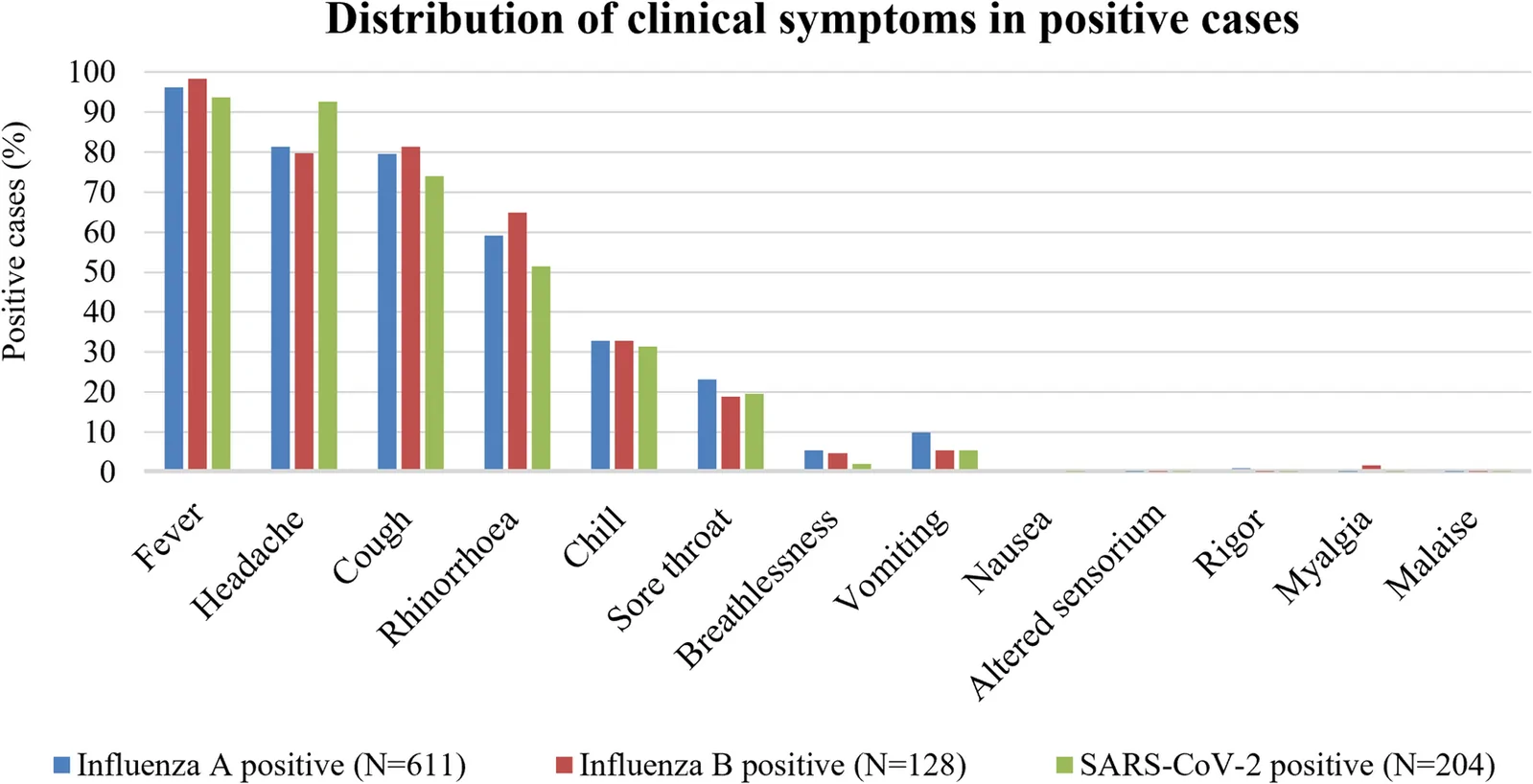
Representation of symptoms in positive cases of Influenza A, Influenza B and SARS-CoV-2. The symptoms represented by the study subjects were categorized for all the three viruses from January 2025-August 2025. Overall, 12.3% (611/4948) were positive for Influenza A, 2.6% (128/4948) were positive for Influenza B, and 4.1% (204/4948) were positive for SARS-CoV-2.

**Fig. 2:**
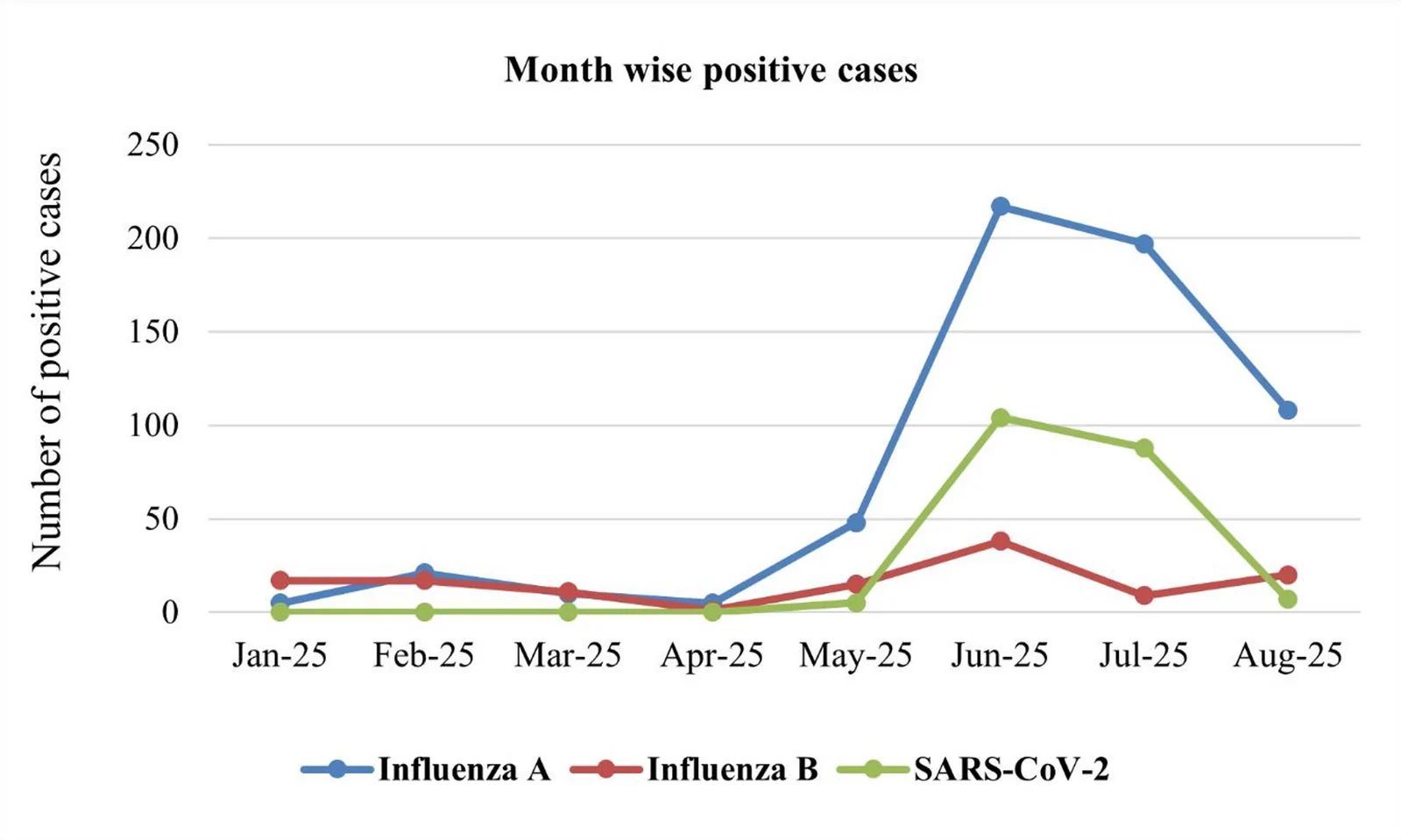
Month wise distribution of positive cases of Influenza A, Influenza B and SARS-CoV-2. Figure shows the positive count for all the three viruses month-wise from January 2025 to August 2025. During the period, 12.3% (611/4948) were positive for Influenza A, 2.6% (128/4948) were positive for Influenza B, and 4.1% (204/4948) were positive for SARS-CoV-2.

While comparing the symptoms with respect to all three viruses, it was found that the symptom trends observed in Influenza (both A and B) along with SARS-CoV-2 positive cases showed a consistent clinical pattern across all three pathogens except for myalgia, which was not found to be associated with the Influenza A positive cases. Figure 1 shows the representation of symptoms in positive cases of Influenza A, Influenza B and SARS-CoV-2. The monthly prevalence rate of all three viruses was presented in Figure. 2. The Pearson Chi-square test showed significant temporal variation across the months for Influenza A (*p* < *0.001*), Influenza B (*p* *<0.001* ) and SARS-CoV-2 (*p* <*0.001*). While Influenza A and B were detected consistently from January 2025 through August 2025, a marked increase in case numbers was noted from May 2025 onwards. The monthly prevalence trend showed that the Influenza A gradually increased, reaching a peak in June 2025 to July 2025 (20%, for June and 27.2% for July). Influenza B showed the highest prevalence in June 2025 (3.5%). It was evident from our data that the resurgence of COVID-19 began in May 2025, culminating in 197 confirmed cases over the May 2025-July 2025 period showing 0.8% for May, 9.6% for June, and 12.2% for July respectively, as presented in Fig. 2. The count decreased in August with 1.5% prevalence (Fig. 2). It is noteworthy to mention that during this period, our surveillance program was consistent in terms of stable testing capacity and sampling practices. The study had no changes in case definitions or healthcare-seeking patterns across sites. While minor variations in health-seeking behaviour cannot be ruled out, the increased prevalence of symptomatic cases during May to July likely represents a genuine rise in virus circulation.

### Co-infection cases

A total of two co-infection cases were detected in the study period of which one was Influenza A with Influenza B co-infection and the other one was Influenza A with SARS-CoV-2. The clinical and demographic features of both the co-infection cases were presented in supplementary Table 2.

#### Case presentation 1

A 27-year-old female with no significant past medical history presented with influenza-like symptoms (headache, fever, vomiting, body pain, rhinorrhoea, cough, and sore throat) of an almost two-day duration during the 1st week of June, 2025. She was vaccinated for SARS-CoV-2 but not for Influenza. The lady had no medical history of any severe illness in the past. The patient’s family history data revealed that none of her family members or neighbours had similar symptoms or illness. She also clarified that she had no travel history during the period. The patient had no other complications, and the vital signs were within normal limits. The first respiratory sample (nasopharyngeal and throat swab) was collected within 24 h of the onset of symptoms. The sample was processed for the extraction of nucleic acid and real-time RT PCR for the detection of Influenza A, Influenza B, and SARS-CoV-2 simultaneously. The sample tested positive for both Influenza A and Influenza B (Influenza A Ct: 24, Influenza B Ct: 22) and negative for SARS-CoV-2. The internal control was also run along with the virus-specific genes for validation of the PCR run. Further, to confirm and validate the findings, a follow-up sample was collected after one day and processed for molecular testing. Retesting also confirmed the simultaneous infection of both the Influenza viruses A and B (Influenza A Ct: 23, Influenza B Ct: 20).

#### Case presentation 2

A 9-year-old female child in general good health presented with influenza-like symptoms (fever, headache, rhinorrhoea, cough, and sore throat) of almost one-day duration during the 3rd week of June, 2025. She was not vaccinated for Influenza and SARS-CoV-2. The female child had no medical history of any severe illness in the past. The patient’s family history data revealed that none of her family members or neighbours had similar symptoms or illness. Her parents also clarified that she had no travel history during the period. The patient had no other complications, and the vital signs were within normal limits. The respiratory sample (nasopharyngeal and throat swab) was collected within 24 h of the onset of symptoms. The sample was processed for the extraction of nucleic acid and real-time RT PCR for the detection of Influenza A, Influenza B, and SARS-CoV-2 simultaneously. The sample tested positive for Influenza A and SARS-CoV-2 (Influenza A Ct value: 22, SARS-CoV-2 Ct value: 25) and negative for Influenza B. The internal control was also run along with the virus-specific genes for PCR validation. The patient’s family did not give consent for the second sample for further validation and follow-up.

#### Subtyping and lineage identification of the co-infection cases

Both the co-infection cases were processed for Influenza A subtyping using real-time PCR, which confirmed the presence of the H3N2 strain. Notably, subtyping of Influenza A positive cases detected in our laboratory between January 2025 and August 2025 showed predominance of H3N2 strain. This finding was further validated through next-generation sequencing (NGS) of the hemagglutinin (HA) gene in representative H3N2-positive samples along with the co-infection cases. Additionally, the sample co-infected with Influenza B also underwent subtyping, revealing it to be of the Victoria lineage, the predominant circulating strain of Influenza B globally since 2020. Of note, all the Influenza B positive samples from our laboratory during the period were sub-typed as Influenza B: Victoria and no sample with the Yamagata lineage was detected. NGS-based whole genome sequencing confirmed that the SARS-CoV-2 positive co-infection case was associated with the XFG lineage of SARS-CoV-2 Omicron variant, which has been the predominant circulating strain in the country since May 2025.

#### Clinical characteristics of the co-infection cases

Given that all three respiratory viruses transmit through the identical respiratory route, we were prompted to examine the symptomatic differences between the two cases. Clinical data obtained from the patients showed that headache, fever, cough, rhinorrhoea and sore throat were the common symptoms associated with both co-infection cases. Interestingly, the case involving co-infection with both Influenza A and Influenza B exhibited two additional symptoms: vomiting and body ache (Myalgia), which may be attributed to the combined effect of Influenza A and B co-infection in the patient. These symptoms were not observed in the Influenza A and SARS-CoV-2 co-infection case.

### Sequencing and mutational analysis of the co-infection cases

#### Influenza A (H3N2) HA gene sequencing

**Fig. 3:**
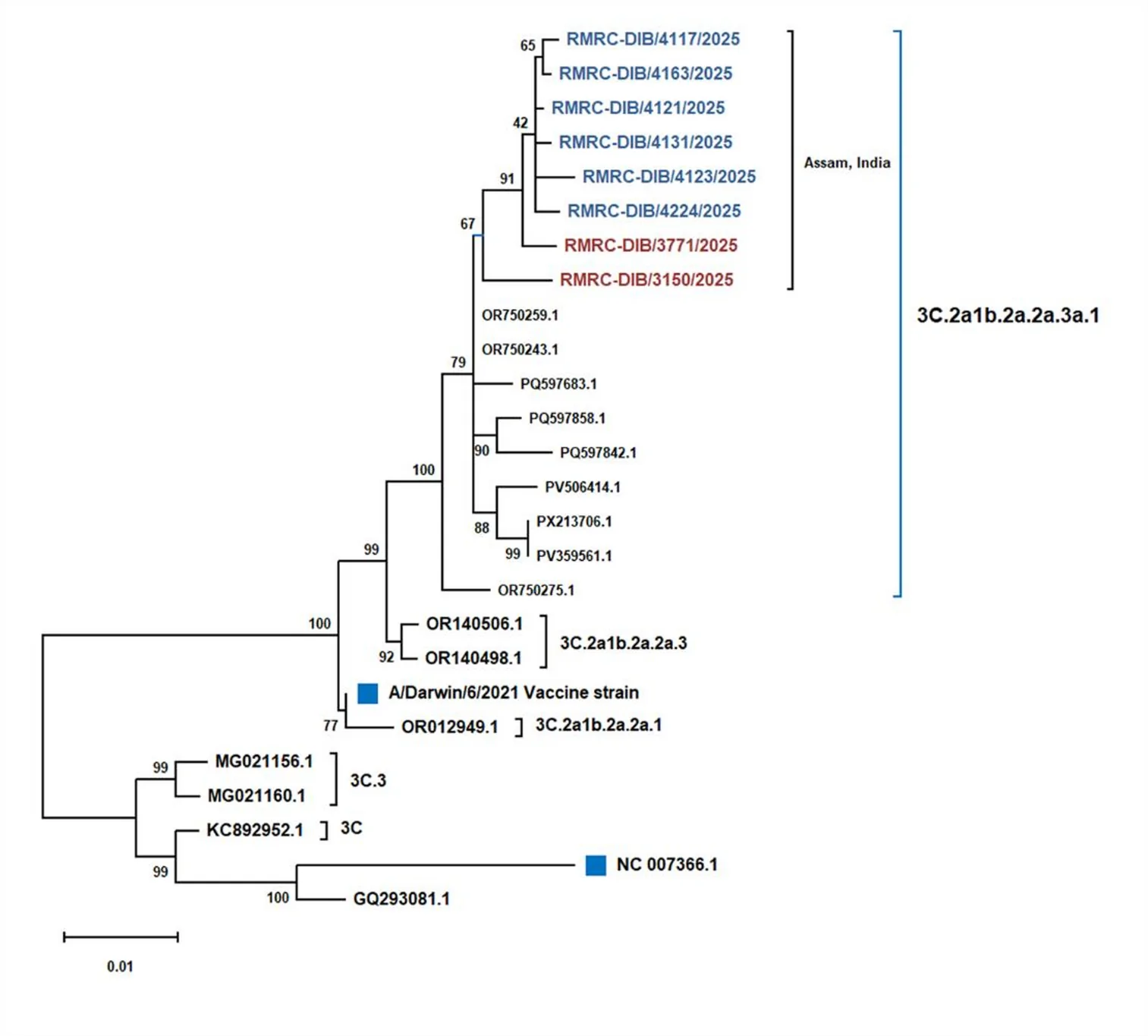
Phylogenetic analysis of Influenza A (H3N2) positive co-infection cases. The phylogenic tree was constructed using the Maximum Likelihood method and General Time Reversible model of nucleotide substitutions and the tree with the highest log likelihood (− 3,463.80) is shown. A total of 500 bootstrap replications has been implemented to enhance the strength of the tree. A total of 26 nucleotide sequences has been included for evolutionary analysis (eight nucleotide sequences from the present study along with 18 representative sequences of globally Influenza A H3N2 strains). Evolutionary analyses were conducted in MEGA X utilizing up to 4 parallel computing threads. A/New York/392/2004 and A/Darwin/6/2021 strains were used as reference strains (marked in blue squares). Six representative Influenza A (H3N2) HA protein sequences from the present study were marked in blue colour and the sequences from the co infection cases from Dibrugarh, Assam were denoted by red colour.

Post sequencing analysis using the Nextclade tool indicated that both Influenza A (H3N2) positive cases belonged to clade 3C.2a1b.2a.2a.3a.1, which was further confirmed by the phylogenetic analysis. The average coverage percentage for the HA gene was found to be 70–80% as per ≥ 30X criteria. The phylogenetic tree was constructed using the Maximum Likelihood method and the General Time Reversible model as presented in Fig. 3. The tree with the highest log-likelihood value (− 3,463.80) was selected. To determine the reliability of the designed tree, 500 bootstrap replications were considered. MEGA X utilising up to 4 parallel computing threads was used for Evolutionary analysis. The constructed phylogenetic tree was comprised of twenty-six sequences with eight sequences from Dibrugarh, Assam, including the co-infection cases (Fig. 3). As shown in the Fig. 3, the eight sequences of Influenza A (H3N2) positive samples from the present study clustered under the clade 3C.2a1b.2a.2a.3a.1 along with nine globally circulating H3N2 strains from 2023 to 2025. A/New York/392/2004 and A/Darwin/6/2021 strains were used as reference strains (marked in blue squares). Six representative Influenza A (H3N2) HA protein sequences from the present study were marked in blue colour and the sequences from the co-infection cases were denoted by red colour (Fig. 3). The datasets generated and analysed during the current study were submitted to the GISAID repository and accession numbers are: EPI_ISL_20158394, EPI_ISL_20158389, EPI_ISL_20158390, EPI_ISL_20158391, EPI_ISL_20158393, EPI_ISL_20158398, EPI_ISL_20158401, EPI_ISL_20158417. Although a total of 33 samples were processed for Influenza HA sequencing, these eight samples were selected based on the viral load, sequencing quality and coverage (1.4–1.6 kb) of the HA protein.

**Fig. 4:**
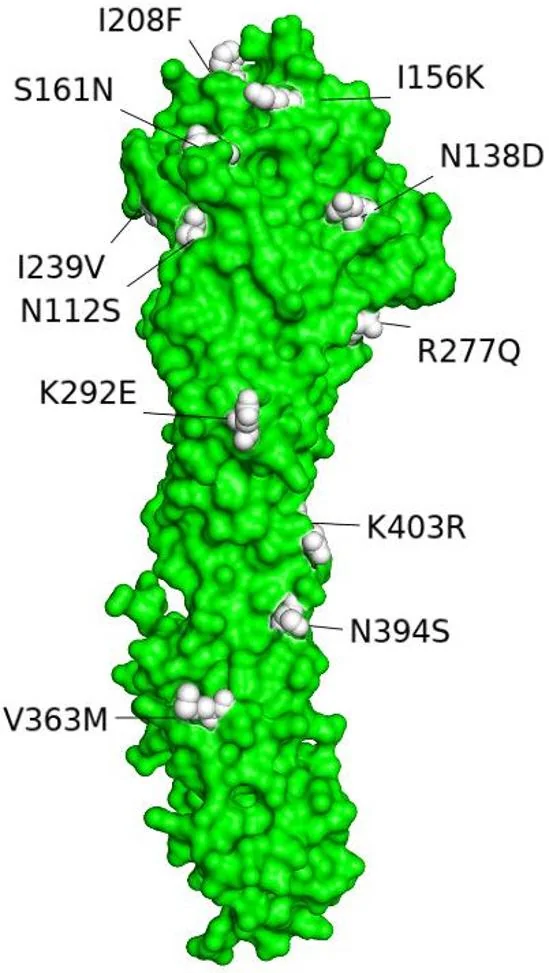
HA protein mutations of the co-infection cases against the vaccine strain. Structure of the H3N2 HA monomer showing amino acid substitutions in the studied co-infection case (EPI_ISL_20158389) as compared to A/Darwin/6/2021 vaccine strain. The Crystallographic structure of the HA protein was predicted using Swiss Model server. The observed mutations were visualized in PyMOL (v.2.5.5.). Overall HA protein structure was denoted by green colour while the mutations were marked with white colour.

Mutational analysis was performed on the MEGA X software for the detection of the HA-specific mutations in comparison to the reference vaccine strain A/Darwin/6/2021 (accession number: OQ718999.1). All the representative HA specific mutations observed in the co-infection cases, along with other representative sequences are presented in Table 2. The multiple sequence alignment of the hemagglutinin (HA) gene sequences from the study samples is presented in supplementary Fig. 1 highlighting the observed amino acid substitutions. It is important to add here that several amino acid substitutions were observed in the co-infection cases which were absent in the reference vaccine strain. In contrast, mutations including S161N, R277Q, V363M and K403R were only associated with the Influenza A and Influenza B co-infection case and absent in all other studied H3N2 HA sequences, including the vaccine strain, A/Darwin/6/2021. The amino acid substitution of T151K was surprisingly absent in the Influenza A and Influenza B co-infection case, along with the vaccine strain, as presented in Table 2. Notably, T151K present in most study sequences lies within antigenic site A, a major neutralising antibody-binding region. This mutation has been associated with reduced antibody reactivity and potential vaccine mismatch as per earlier reports, suggesting its emergence may contribute to immune escape^23^. R277Q mutation is also found to be associated with the newly linked N-glycosylation sites, thus affecting antibody binding^24^. The mutational analysis data from the present study thus hinted that the circulating H3N2 viruses in Northeast India may be undergoing antigenic evolution compared to the current vaccine strain A/Darwin/6/2021. However, the observed mutations may be incidental and functional validation is required before attributing them to co-infection dynamics. The HA protein crystallographic structure showing the HA-specific mutations detected in the present study is presented in Fig. 4. The dN/dS ratio was comparatively low (≤ 1) in the HA protein of the Influenza co-infection cases, hinting the negative selection.

**Table 2:** HA specific amino acid mutations in the Influenza A (H3N2) positive cases against the vaccine strain.

Sample ID	*N* 112 S	*N* 138 D	T 151 K	I 156 K	S 161 *N*	K 205 *R*	I 208 F	I 239 V	*R* 277 Q	K 292 E	V 339 I	V 363 M	*N* 394 S	K 403 *R*
**A/Darwin/6/2021**	A	A	A	A	A	A	A	A	A	A	A	A	A	A
**RMRC-DIB/3150/2025**	P	P	A	P	P	A	P	P	P	P	A	P	P	P
**RMRC-DIB/3771/2025**	P	P	P	P	A	A	P	P	A	P	P	A	P	A
RMRC-DIB/4117/2025	P	P	P	P	A	P	P	P	A	P	A	A	P	A
RMRC-DIB/4121/2025	P	P	P	P	A	P	P	P	A	P	A	A	P	A
RMRC-DIB/4123/2025	P	P	P	P	A	P	P	P	A	P	A	A	P	A
RMRC-DIB/4131/2025	P	P	P	P	A	P	P	P	A	P	A	A	P	A
RMRC-DIB/4163/2025	P	P	P	P	A	P	P	P	A	P	A	A	P	A
RMRC-DIB/4224/2025	P	P	P	P	A	P	P	P	A	P	A	A	P	A

In the table, each row corresponds to an individual Influenza A (H3N2) positive sample marked with sample ID including the reference vaccine strain (A/Darwin/6/2021) and each column shows the specific amino acid mutations. The presence of the mutation is denoted by ‘P’ and absence is denoted by ‘A’. Sample IDs marked in bold indicate the co-infection cases and the reference vaccine strain.

The genome sequencing could not be completed for Influenza B (Victoria) in the Influenza A with Influenza B co-infection case thus limiting the data interpretation.

#### SARS-CoV-2 genome sequencing

BLAST analysis by comparing the SARS-CoV-2 target sequence from the co-infection case with the original Wuhan strain (Wuhan-Hu-1/2019) confirmed the sequence similarity with the globally circulating SARS-CoV-2 lineages. Further, Nextclade-based analysis showed that the genomic sequence from the co-infection case was placed under clade 25C and lineage XFG of the SARS-CoV-2 Omicron variant. Additionally, mutational analysis using the Nextclade tool identified several mutations in the co-infection sample, including the spike protein like N764K, T572I, D614G, P621S, H655Y, N679K, S939F, Q954H, P1143L, S50L, V127F, G142D, E554K, A570V, S31P and P681R when compared to the reference Wuhan strain. Due to low sequence coverage of SARS-CoV-2 in the sample, the RBD domain could not be properly analyzed resulting in the detection of less number of mutations. The limited high-confidence coverage of SARS-CoV-2 in critical regions restricted the detailed mutational interpretation. This is one of the limitations of our study. The obtained sequence of SARS-CoV-2 was submitted to the GISAID repository (accession number: EPI_ISL_20179540). For SARS-CoV-2 average coverage depth was observed to be approximately 357 X. However, the average RBD percentage was 88.7% overall and 8.5% as per ≥ 30X criteria.

### Presence of N-glycosylation sites in the HA protein of Influenza A (H3N2) strains

**Fig. 5:**
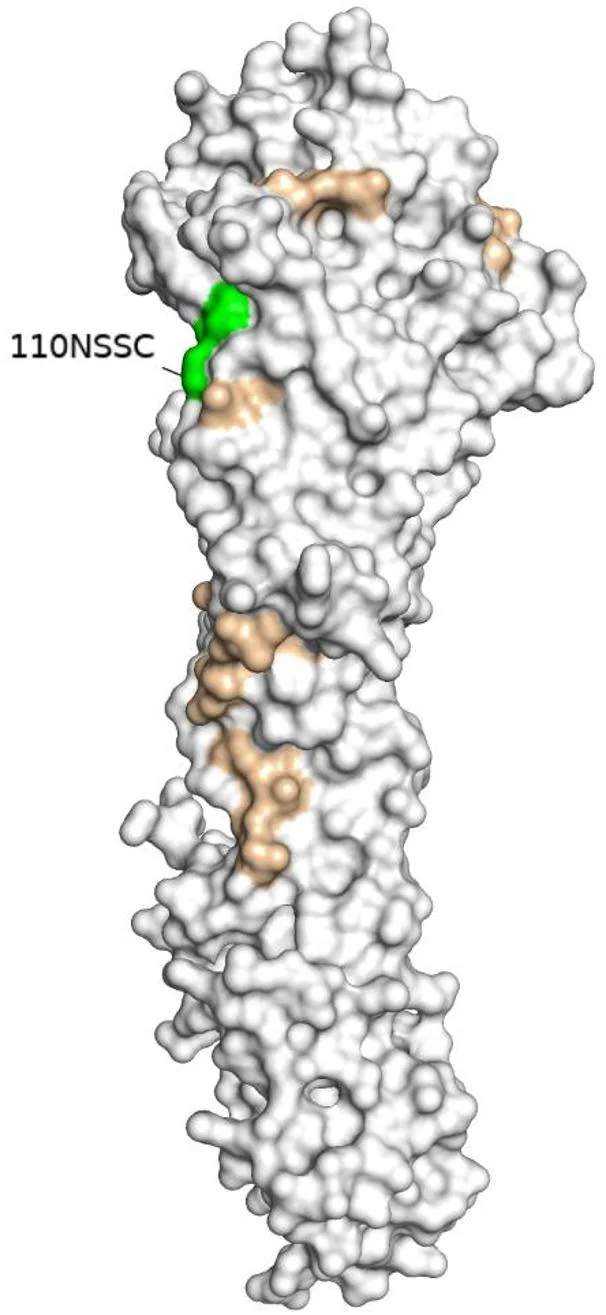
N-glycosylation sites observed in the co-infection cases in comparison to vaccine strain. The glycosylation sites were predicted using the online NetNglyc server 1.0. The analysis was performed using default parameters, and those sites with prediction scores exceeding the default threshold of 0.5 were considered as potential glycosylation sites. The green colour represented newly acquired N-glycosylation site at position 110 of HA protein. N-glycosylation sites common with A/Darwin/6/2021 were shown in the wheat colour.

The prediction of the HA protein-based N-glycosylation sites of Influenza A (H3N2) co-infection cases showed that sequon (N X-T/S) detected at sites 79, 181, 262, and 301 in both the co-infection cases, along with the other target sequences, were conserved as compared to the reference strain. However, it is worth mentioning that a newly acquired N-glycosylation site at position 110 was detected at the HA protein of Influenza A (H3N2) co-infection cases, which was absent in the reference vaccine strain. Interestingly, the co-infection case with influenza A and B showed additional preserved N-glycosylation sites at positions 54, 61, and 149 as compared to the H3N2 vaccine strain. The findings suggest that the observed glycosylation patterns may influence ongoing viral evolutionary changes in the co-infection cases. In the Fig. 5, the glycosylation sites were visualised in the crystal structure of the HA protein. The newly acquired N-glycosylation site at position 110 was denoted by the green colour, and conserved sites were marked with wheat colour (Fig. 5).

## Discussion

The study provides one of the first detailed clinical and genomic descriptions of respiratory viral co-infections from Northeast India, documenting co-infections of Influenza A (H3N2) with SARS-CoV-2 and Influenza A (H3N2) with Influenza B (Victoria). Both cases have been identified through routine surveillance of suspected ARI and paediatric SARI cases conducted in Dibrugarh, Assam, India, between January 2025 and August 2025. Multiple previous studies have reported cases of co-infections, including one triple infection involving both Influenza viruses A and B together with SARS-CoV-2^13,17^. It is generally believed that co-infections are associated with poor clinical outcomes and increased severity, with particular emphasis on co-infections of SARS-CoV-2 and Influenza viruses^13,25,26^. However, in the present study, both the co-infection cases had recovered within one week without hospital admission. Our findings were in congruence with previous reports where authors documented that no severe disease was associated with Influenza A co-infection, either with SARS-CoV-2 or Influenza B^14,27^. When analyzing the symptoms of the co-infection cases, it was found that headache, fever, cough, rhinorrhoea, and sore throat were commonly observed in both co-infection cases. However, the case involving co-infection with Influenza A and Influenza B presented additional symptoms, including vomiting and body pain, consistent with findings from a previous study^13^. It is important to highlight that the ARI/SARI surveillance carried out in this study identified co-infection cases in only 0.04% (2/4948) of samples, indicating that co-infections are uncommon and represent a rare occurrence^28^. However, it was reported that co-infection of Influenza A with SARS-CoV-2 was higher in comparison to infection with Influenza B (Victoria). The reason behind this might have been that Influenza A had a broader host range with the ability to undergo frequent mutations and large seasonal coverage than Influenza B. These might serve as the contributing factors for the co-infections during the peak flu period^29^. Several studies reported that Influenza virus infection rises in the summer/monsoon months (June-August) in tropical and subtropical settings^30,31^ A multi-country analysis from southern and south-eastern Asia had shown that Influenza positivity often reached its peak between June and October in several countries, including India which is consistent with our findings^30^.

Genomic analysis revealed that all circulating H3N2 viruses in this study belonged to clade 3C.2a1b.2a.2a.3a.1, consistent with contemporary global circulation patterns^32,33^. Similarly, the SARS-CoV-2 sequence from the co-infection case have been classified within the XFG lineage of SARS-CoV-2 Omicron variant, corresponding to clade 25C in agreement with the latest WHO lineage designations. Given the propensity of Influenza A to undergo frequent conformational and mutational changes primarily in the hemagglutinin (HA) region, our study further examined whether the co-infection cases exhibited any unique or altered amino acid mutations with the studied reference stain. Specifically, mutations were compared against the reference sequence and sequences obtained from single infections of Influenza A (H3N2) to assess any potential differences attributable to co-infection. The detection of multiple amino acid substitutions in our study relative to the A/Darwin/6/2021 vaccine strain highlights the ongoing evolution of H3N2 in the region, which agrees with the previous findings^34,35^. It is noteworthy to mention that the T151K mutation was present on the HA sequence of the Influenza A (H3N2) and SARS-CoV-2 co-infection case along with the representative HA sequences of the studied samples, but absent in the Influenza A and Influenza B co-infection case. A molecular characterization study from Thailand during 2018-19 detected T151K mutation in the HA protein of the Influenza A (H3N2) isolates of patients. The T151K substitution is located within the globular head of hemagglutinin in close proximity to the 150-loop (the receptor-binding site, and antigenic site A). This mutation is characterized by the replacement of threonine with lysine, a positively charged amino acid that is likely to alter the local electrostatic landscape and modulate loop conformation adjacent to the receptor-binding area. Such changes may affect the geometry of sialic acid engagement and potentially trigger the receptor binding and antigenicity^36,37^. T151K plays an important role in antigenicity may be potentially impacting viral recognition by the immune system and influencing vaccine match or immune escape^23^. Similarly, the R277Q substitution occurs in an antigenically exposed region of the globular head of HA1 outside the core receptor-binding area. Arginine normally contributes to local structural stability through salt bridge formation and its replacement with the neutral, polar glutamine may disrupt stabilizing electrostatic interactions by reducing local charge^38,39^. R277Q is found to be associated with the newly linked N-glycosylation sites, thus may be affecting binding of the antibodies and antigenicity^24^. The previously published studies mentioned that T151K and R277Q mutations present on antigenic epitopes A and C and may contribute towards emerging drug resistance and antigenic drift. Hence, continuous genomic surveillance and molecular screening are needed for restricting poor clinical outcomes^23,36^. Although the presence of some of these mutations were documented previously, till date, there are no reports on the association of these mutations with Influenza or SARS-CoV-2-associated co-infection cases^23,40^. Similarly, the V339I amino acid substitution is only found to be associated with the Influenza A and SARS-CoV-2 co-infection case. In support to our findings, a previously published study from Myanmar showed the presence of amino acid substitutions like S112N, R277Q, V363M, and T151K in comparison to the vaccine strain A/Switzerland/8060/2017^40^. It is essential to mention here that there is limited literature on the mutational analysis studies of Influenza A (H3N2) highlighting the mutations like S161N, V363M, K403R, and V339I, thus accentuating the detailed genomic and structural analysis of the circulating H3N2 strains against the vaccine strains.

In this study, HA mutations observed in the co-infection cases were compared primarily with the A/Darwin/6/2021 vaccine strain. Further, a comparative analysis of the HA gene mutations was performed by taking five representative 2025 Influenza A (H3N2) HA sequences from GISAID along with the reference strain and our study sequences (supplementary Fig. 1). These globally sourced reference sequences provide a representative snapshot of the contemporary genetic diversity of circulating H3N2 viruses together with the sequences from our samples.

Glycosylation of hemagglutinin (HA) and neuraminidase (NA) proteins occurs via an oligosaccharide adhering to the side-chain amide nitrogen of an asparagine (Asn) residue within the conserved N-linked glycosylation (NLG) motif: Asn-X-Ser/Thr, X denotes any amino acid excluding proline^41^. In our study, N-glycosylation sites identified in the H3N2 sequences including co-infection cases, specifically at HA positions 79, 181, 262, and 301, are consistent with previous reports^41–43^. An interesting finding of our study is the detection of the NLG site at position 110 of the HA protein in Influenza A (H3N2) co-infection cases, which was absent in the studied reference vaccine strain. Consistent with our findings, earlier studies also reported the absence of these glycosylation sites in earlier vaccine strains may indicate a potential evolutionary trend^42,44^. Interestingly, the co-infection case of influenza A with B showed additional preserved N-glycosylation sites at positions 54, 61, and 149 in reference to the H3N2 vaccine strain, substantiating the notion that H3N2 co-infections (including A/B co-infections) have acquired additional HA glycosylation sequences, which might contribute to the structural changes in the genome. One previously published study reported that the HA globular head is immunodominant, containing five major antibody-binding sites where amino acid substitutions can diminish antibody recognition^44^. Studies have also hypothesized that N-linked glycosylation can influence antigenicity depending on its structural location on HA^41,43^. However, the actual impact of the sites observed here cannot be inferred without targeted functional assays. Hence, these findings should be interpreted cautiously serving primarily as indicators of molecular variability among circulating strains.

The present study has several limitations. The study detected only two cases of co-infection with a 0.04% prevalence (2/4948), which is a major limitation of the study. Further, no follow-up sample could be collected for the Influenza A and SARS-CoV-2 co-infection case for better case presentation. HA gene-based targeted sequencing was performed instead of whole-genome sequencing for Influenza A (H3N2) positive cases. Another limitation of this study is the low sequence coverage of SARS-CoV-2 in the co-infection case, which prevented detailed analysis of the RBD region and likely reduced the number of detectable mutations from the region. Further, Sequencing could not be performed for Influenza B (Victoria) in the Influenza A with Influenza B co-infection case. In addition, the present study has not performed functional assays for validation of biological relevance. Future studies with higher sequencing coverage and inclusion of whole-genome sequencing for all co-detected viruses will be crucial to fully understand potential interactions between co-infecting viruses. Although co-infection cases in this study did not show increased clinical severity, their genomes exhibited clear divergence from both the vaccine strain and regional single-infection sequences. These findings underscore the importance of continued genomic and clinical surveillance to monitor viral evolution, detect antigenic drift, and inform regional vaccine strain selection from the Northeast region.

In conclusion, the present study from Northeast India provides important insights into co-infections involving Influenza A (H3N2) with SARS-CoV-2, and Influenza A (H3N2) with Influenza B (Victoria) by incorporating demographic, clinical and genomic analysis. A total of 4,948 cases were enrolled with two co-infection cases detected. This preliminary report suggests that co-infections may not always be related to clinical severity but could influence viral evolution through marked genomic differences. The detection of mutations and glycosylation changes compared to the vaccine strain emphasizes the need for ongoing genomic surveillance and specific, hypothesis-driven follow-up studies to better understand emerging mutations and the development of future vaccine strategies.

## Methods

### Design of the study, study site and cases

The study was conducted at the Regional VRDL, ICMR-RMRCNE, Dibrugarh, Assam, between 1st January 2025 and 31st August 2025. The respiratory swab samples (Nasopharyngeal and throat) were collected in Viral Transport Medium (VTM) from patients presenting with suspected respiratory infections at neighbouring hospitals in Dibrugarh, Assam. Enrolment of the patients was performed following the WHO case definitions for Influenza-like Illness (ILI), ARI, and SARI. The study was ethically approved at ICMR-RMRCNE, and all procedures were conducted as per the ethical standards set by the Ethics Committee of the Institute (letter No.RMRC/Dib/IEC (Human)/2022-23/3031, date: 02/01/2024).

### Demographic and clinical data collection

All the relevant data (demographic and clinical) of the patients have been collected in the standard format of the case request Form (CRF) available in the laboratory. Trained technicians obtained relevant information directly from patients during field visits for sample collection and recorded it in the CRFs. Cases identified with co-infection were selected for follow-up, and additional data were gathered through telephonic interviews conducted in the native language. The patients were questioned on symptoms, duration of illness, vaccination status on Influenza and COVID-19, presence of co-morbidities, any complications and hospitalization details. For the minor patient with co-infection, interview was conducted with parents or legal guardians.

### Real-time PCR-based molecular detection and sub-typing

The respiratory swab samples were processed for RNA extraction using the MagMax nucleic acid extraction kit (Thermo Scientific, USA). The extracted nucleic acid samples were further used for the simultaneous detection of SARS-CoV-2, Influenza A and B using the inhouse primer probes for Influenza A, Influenza B and SARS-CoV-2 and one-step master mix (Takara Bio, USA) as per the manufacturer’s instruction. The reaction mix was comprised of 10.0 µl of PCR mix, 0.4 µl of Ex Taq, 0.4 µl RT enzyme, 2.5 µl of primer-probe mix, 1.7 µl of Nuclease-free water and 5 µl of the template RNA. PCR cycle conditions included: 42 °C for 5 min for reverse transcription, 95 °C for 10 s for hold stage, followed by 40 cycles of PCR amplification stages with 95 °C for 5 s and 60 °C for 34 s. The results obtained were further validated by an ICMR approved multiplex real-time PCR kit (ICMR-NIV multiplex combo kit, lot number:02). This single tube multiplex Influenza and SARS-CoV-2 real-time RT-PCR detection kit contained TaqMan RT-PCR primer probe mix for the qualitative detection and characterization of Influenza A, Influenza B and SARS-CoV-2. The reaction mix was prepared with 5.0 µl of PCR mix, 1 µl of primer-probe mix, 7 µl of nuclease free water and 7 µl of template RNA. PCR conditions included: 50 °C for 5 min for reverse transcription, 95 °C for 20 s for hold stage followed by 40 cycles of PCR amplification stages with 95 °C for 5 s and 55 °C for 30 s. The cut-off Ct value was decided as in the range of 15–25 for the positive control and the Ct value *≤* 36 for each of the samples. The cases tested positive for Influenza (A or B) were processed for sub typing and lineage identification using real-time PCR by the ICMR-NIV Influenza sub typing kit. The reaction mix was prepared with 5.0 µl of PCR mix, 1 µl of primer-probe mix, 7 µl of nuclease free water and 7 µl of template RNA. PCR conditions included: 53 °C for 10 min for reverse transcription, 95 °C for 2 min for polymerase activation followed by 40 cycles of PCR amplification stages with 95 °C for 3 s and 55 °C for 30 s. The identified co-infection cases were processed for NGS-based sequencing of SARS-CoV-2 and Influenza A (H3N2).

### NGS and post-sequencing data analysis

NGS was performed in an Illumina MiSeq system with a 2 × 75 paired-end v3 Flow Cell using the COVIDSeq assay as per the manufacturer’s protocol (Illumina, CA, USA). The targeted HA gene sequencing (1.7 kb approx) was employed for Influenza A (H3N2) positive samples, while the SARS-CoV-2 positive co-infection case was processed for SARS-CoV-2 whole-genome sequencing using Illumina COVIDSeq kit. NGS data analysis was conducted using the Linux Command Line Interface (CLI). Read quality was assessed with fastp (v0.23.2)^45^ which filtered and corrected low-quality reads. High-quality reads were then aligned to the reference genomes (NC_045512.2 for SARS-CoV-2, OQ718999.1 for Influenza A: H3N2) using the Bowtie2 aligner (v2.5.2)^46^. The resulting binary alignment files were sorted and indexed with Samtools (v1.19)^47^ prior to consensus generation. Consensus sequences were generated using iVar (v1.4.2)^48^ and subsequently used for downstream analysis. Additionally, variant analysis was performed using the Nextclade online tool (https://clades.nextstrain.org/)*.*

### Phylogenetic analysis, mutational analysis, and prediction of glycosylation sites

The target sequences from co-infection cases were analysed against closely associated complete reference genomes alongside the nearest available sequences from NCBI. Sequence similarity searches were conducted using the BLAST tool available on the NCBI website to identify the most closely related sequences. To estimate evolutionary divergence, pairwise distance calculations were performed using the Maximum Composite Likelihood model including rate variation among sites and a gamma distribution model. Furthermore, the data were examined for non-synonymous (dN) and synonymous (dS) mutations. Determination of the dN/dS ratio was performed using the Nei–Gojobori method in MEGA X software^49^. For the phylogenetic analysis of the Influenza A(H3N2) cases, representative HA sequences were downloaded from the NCBI database (https://www.ncbi.nlm.nih.gov/). These downloaded sequences, along with the vaccine strain and selective sequences from our study  (including sequences from the co-infection cases) were considered for the phylogenetic analysis of Influenza A (H3N2). The multiple sequence alignment of HA sequences was performed using ClustalW in MEGA X (Version 11).

The phylogenetic tree was constructed using the Maximum Likelihood method based on the General Time Reversible (GTR) model of nucleotide substitution^50^. The tree with the highest log-likelihood value (− 3,463.80) is presented. To assess the reliability of the inferred tree, 500 bootstrap replications were performed. A total of twenty-six nucleotide sequences were included in the evolutionary analysis, comprising eight sequences obtained in the present study and eighteen representative sequences of globally circulating Influenza A (H3N2) strains. Evolutionary analysis was conducted in MEGA X utilising up to 4 parallel computing threads.

The aligned HA gene amino acid sequences of the co-infection cases and the representative single infection cases against the vaccine strain A/Darwin/6/2021 (accession number: OQ718999.1) were analysed for amino acid mutations. The HA gene-specific amino acid mutations were determined. For the determination of the N-glycosylation sites linked to the HA protein, the online NetNglyc server 1.0 was used. The analysis was performed using default parameters, and those sites with prediction scores exceeding the default threshold of 0.5 were considered as potential glycosylation sites. The obtained data were further validated with the Nextclade tool.

In the case of SARS-CoV-2, the genome sequence was compared with the Wuhan reference strain (Wuhan-Hu-1/2019, accession number: NC_045512.2). The amino acid mutations were determined in the Nextclade online tool by comparing with the above-mentioned reference sequence.

### Statistical analysis

Statistical analysis was performed to determine the relationship between demographic and clinical characteristics with the virus positivity. Categorical data were expressed as counts and percentages. Group differences were evaluated using Pearson’s chi-square test or Fisher’s exact test. Odds ratios with 95% confidence intervals were calculated from the contingency tables. Missing data were excluded from the analysis. All analyses were two-tailed and a *p* < 0.05 was considered as statistically significant. SPSS version 22.0 and MedCalc software were used for data analysis.

## Supplementary Information

Below is the link to the electronic supplementary material.

Supplementary Material 1

Supplementary Material 2

Supplementary Material 3

## Data Availability

The datasets generated and analysed during the current study are available in the GISAID repository and accession numbers are: EPI_ISL_20158394, EPI_ISL_20158389 EPI_ISL_20158390, EPI_ISL_20158391, EPI_ISL_20158393, EPI_ISL_20158398 EPI_ISL_20158401, EPI_ISL_20158417 (Influenza A) and EPI_ISL_20179540 (SARS-CoV-2). The sequence files downloaded from GISAID for the Influenza A (H3N2) HA segment and the SARS-CoV-2 sequences submitted from this study were provided as supplementary materials 1 and 2 (gisaid_epiflu_sequence_Influenza.doc and gisaid_hcov-19_SARS-CoV-2 sequence.doc).
