# Supplementary material for: Clinical and genomic characterization of Influenza A co-infection with SARS-CoV-2 and Influenza B: a respiratory surveillance study in Assam, India
Source: Sci Rep. 2026 Jun 12;16:27900. doi: 10.1038/s41598-026-55080-0 (PMC13547378; doi:10.1038/s41598-026-55080-0)
Supplement: Supplementary file 1 [file 41598_2026_55080_MOESM1_ESM.docx]

| **Major symptoms** | **Symptomatic Status** | **Total N** | **Influenza A positive**  **N (%)** | **Influenza A negative**  **N (%)** | **Odds ratio** | **95% CI** | **P value** |
| --- | --- | --- | --- | --- | --- | --- | --- |
| Fever | Yes | 4214 | 587 (13.9) | 3627 (86.1) | 5.03  1.0 | 3.26-7.76  Ref | <0.001 ^Ψ^ |
|  | No | 706 | 22 (3.1) | 684 (96.8) |  |  |  |
| Cough | Yes | 3705 | 482 (13) | 3223 (87) | 1.28  1.0 | 1.04-1.57  Ref | 0.01 ^Ψ^ |
|  | No | 1214 | 127 (10.5) | 1087 (89.5) |  |  |  |
| Headache | Yes | 3458 | 495 (14.3) | 2963 (85.7) | 1.977  1.0 | 1.59-2.44  Ref | <0.001 ^Ψ^ |
|  | No | 1463 | 114 (7.8) | 1349 (92.2) |  |  |  |
| Rhinorrhoea | Yes | 2925 | 356 (12.2) | 2569 (87.2) | 0.954  1.0 | 0.583-0.803  Ref | 0.312 ^Ψ^ |
|  | No | 1995 | 253 (12.7) | 1742 (87.3) |  |  |  |
| Sore throat | Yes | 1311 | 141 (10.8) | 1170 (89.2) | 0.809  1.0 | 0.66-0.98  Ref | 0.02 ^Ψ^ |
|  | No | 3609 | 468 (13) | 3141 (87) |  |  |  |
| Breathlessness | Yes | 507 | 32 (6.3) | 475 (93.7) | 0.448  1.0 | 0.31-0.64  Ref | <0.001 ^Ψ^ |
|  | No | 4413 | 577 (13.1) | 3836 (86.9) |  |  |  |

**Supplementary Table 1: Major symptoms with respect to Influenza A virus infection**

p-value <0.05 considered significant, ^Ψ^ Based on Fisher’s Exact Test. Reference category is indicated by ‘Ref’
