# Supplementary material for: Clinical and genomic characterization of Influenza A co-infection with SARS-CoV-2 and Influenza B: a respiratory surveillance study in Assam, India
Source: Sci Rep. 2026 Jun 12;16:27900. doi: 10.1038/s41598-026-55080-0 (PMC13547378; doi:10.1038/s41598-026-55080-0)
Supplement: Supplementary file 2 [file 41598_2026_55080_MOESM2_ESM.docx]

| **S. No** | **Patient type** | **Age**  **(years)** | **Gender** | **Duration of illness** | **Symptoms** | **Diagnosis** | **Detected Ct value** | **Hospital admission** | **Vaccine** | **Co-morbidities** | |
| --- | --- | --- | --- | --- | --- | --- | --- | --- | --- | --- | --- |
| 1 | OPD | 9 | Female | 6 days | Headache, Fever, rhinorrhea, cough and sore throat | Influenza A with SARS-CoV-2 | Influenza A: 22  SARS-CoV-2: 25 | No | SARS-CoV-2: No  Influenza: No | | None |
| 2 | OPD | 27 | Female | 5 days | Headache, fever, vomiting, myalgia, rhinorrhea, cough and sore throat | Influenza A with Influenza B | Influenza A: 24  Influenza B: 22 | No | SARS-CoV-2: Yes  Influenza: No | | None |

**Supplementary Table 2: Clinical and demographic representation of the co-infection cases**
